# Pathological Significance of GLUT-1 Expression in Breast Cancer Cells in Diabetic and Obese Patients: The French Guiana Study

**DOI:** 10.3390/cancers14020437

**Published:** 2022-01-16

**Authors:** Valentin Suteau, John Bukasa-Kakamba, Beatrice Virjogh-Cenciu, Antoine Adenis, Nadia Sabbah, Kinan Drak Alsibai

**Affiliations:** 1 Department of Pathology, Cayenne Hospital Center André Rosemon, F-97306 Cayenne, French Guiana; valentin.suteau@ch-cayenne.fr; 2 Department of Endocrinology and Metabolic Diseases, Cayenne Hospital Center André Rosemon, F-97306 Cayenne, French Guiana; john.bukasakakamba@ch-cayenne.fr (J.B.-K.); nadia.sabbah@ch-cayenne.fr (N.S.); 3 Department of Medicine, Hôpital de jour Adults, Cayenne Hospital Center André Rosemon, F-97306 Cayenne, French Guiana; beatrice.cenciu@ch-cayenne.fr; 4 Clinical Investigation Center Antilles French Guiana (CIC INSERM 1424), Cayenne Hospital Center André Rosemon, F-97306 Cayenne, French Guiana; antoine.adenis@ch-cayenne.fr; 5 Center of Biological Resources (CRB Amazonie), Cayenne Hospital Center André Rosemon, F-97306 Cayenne, French Guiana

**Keywords:** obesity, type 2 diabetes, breast cancer, GLUT-1, cancer metabolism, French Guiana

## Abstract

**Simple Summary:**

This study describes the clinical, histological, and molecular features of breast cancer in French Guiana, and characterizes the expression of the tumor metabolic marker GLUT-1 in breast cancers cells in diabetic and obese patients compared to a control group. This study reveals an overall overexpression of GLUT-1 in 60% of invasive breast carcinomas and in all medullary pattern and carcinoma in situ lesions. Our results highlight the potential role of GLUT-1 as a tumor metabolic prognostic marker and also as an interesting target therapy, independently of patient metabolic disorder.

**Abstract:**

The prevalence of obesity and type 2 diabetes is higher in French Guiana compared to mainland France. These metabolic disorders are associated with an increased risk of cancer. One of the factors involved is hyperinsulinemia that promotes the action of glucose transporter 1 (GLUT-1). The objective of this study is to characterize the expression of GLUT-1 in breast cancers cells in diabetic and obese patients compared to those who are not and to describe the clinical and histological prognostic factors of breast cancer in this population. We conducted a monocentric study including patients with breast cancer diagnosed between 2014 and 2020. Patients were classified into three groups: diabetes, obesity, and control group. The GLUT-1 expression was assessed by immunohistochemistry. In total, 199 patients were included in this study. The median age was 53.5 years, and the median tumor size was 2.8 cm. Luminal A was the most frequent molecular type (58.1%), followed by the triple-negative type (19.9%). The breast cancer in our population was characterized by a younger age at diagnosis, more aggressive molecular types, and larger tumor size. Thus, we suggest the advancement of the age of breast cancer screening in this territory. A total of 144 patients (31 diabetes, 22 obese, and 91 control group) were included for the study of GLUT-1 expression. Overexpression of GLUT-1 was observed in 60.4% of cases and in all carcinoma in situ lesions. GLUT-1 overexpression was associated with more aggressive cancers. This overexpression is correlated with high histological grade, high proliferation index, and aggressive molecular types. Our study found no difference in GLUT-1 expression between the diabetic or obese patients and the control group. These results highlight the potential role of GLUT-1 as a tumor metabolic prognostic marker and also as an interesting target therapy, independently of patient metabolic disorder.

## 1. Introduction

Obesity and type 2 diabetes are major public health problems in the world, with 600 and 415 million affected adults, respectively [1,2]. These two metabolic disorders are proven risk factors for many cancers, including breast cancer [3]. Since 2020, breast cancer has been the most common cancer in the world [4].

The mechanisms that explain the role of obesity and type 2 diabetes in carcinogenesis are poorly understood. One of the mechanisms is the state of hyperinsulinemia [3]. Hyperinsulinemia could participate in the deregulation of the energetic metabolism of cancers since cancer cells need more energy to proliferate [3,5]. The best-known deregulation mechanism of metabolism is the Warburg effect [6,7], which consists of a redirection of energy production by aerobic glycolysis that requires increased glucose requirements. Glucose transporter 1 (GLUT-1) is the tumor cell’s main means of obtaining glucose [8,9,10]. GLUT-1 is a transmembrane protein that catalyzes the entry of glucose into cells [11] and physiologically ensures the basal level of glucose necessary for their survival [12]. It is overexpressed in many cancers, including lung, esophageal, and breast cancers, and in hepatocellular carcinoma [13]. It has been shown that insulin upregulates the GLUT-1 protein [14]. Insulin leads to an increase in the translation of the mRNA coding for GLUT-1 [14]. The translocation of GLUT-1 transporter to the membrane, its site of action, induces an increase in intracellular glucose [15]. This upregulation of GLUT-1 by insulin is dose-dependent [16]. In addition, inhibition of GLUT-1 results in decreased glucose entry into cancer cells despite insulin, suggesting that GLUT-1 is the primary pathway for insulin-regulated glucose entry [17]. These effects may be partly dependent on the insulin-stimulated PI3K-Akt signaling pathway that regulates GLUT-1 [18,19,20]. Furthermore, preclinical data suggest that GLUT-1 inactivation is a viable therapeutic target and that its inhibition could explain the action of some anti-cancer therapies [10].

French Guiana is a European territory located in South America, with 288,090 inhabitants (Insee 2020) in an area of 86,500 km^2^. Guiana is divided into two zones: a zone mostly covered by primary forest with restricted access where 15% of the population lives and a coastal zone where the three main cities are located; Cayenne, Saint Laurent du Maroni, and Kourou.

The prevalence of metabolic disorders like obesity and type 2 diabetes in French Guiana are higher than in mainland France. The prevalence of diabetes is up to 9.3% in women, and the prevalence of obesity is estimated at 17.9% in adults and 22.1% in women [21]. Thus, type 2 diabetes and obesity are major public health problems in French Guiana. Moreover, breast cancer is the most common cancer in women in this territory [22]. However, breast cancer remains scarcely studied in this territory, particularly its histological, prognostic, and molecular characteristics [23,24].

In order to understand the mechanisms involved in breast cancer in diabetic and obese patients and to look for a potential target therapy, we will investigate whether the hyperinsulinemia state in diabetic or obese patients promotes more frequently GLUT-1 overexpressing cancers. In other words, we will search for a link between patient metabolic disorders and cancer metabolism.

The main objectives of this study are to characterize GLUT-1 expression in breast cancer in diabetic and obese patients compared to non-diabetic and non-obese patients, and to compare histological and prognostic factors between these three groups.

## 2. Materials and Methods

We conducted a monocentric retrospective study between January 2014 and December 2020 at Cayenne hospital Centre Andrée Rosemon (Cayenne, French Guiana, France) including women with a histological diagnosis of invasive breast cancer. This study was carried out using the computerized database of the Department of Pathology (Diamic software). A total of 199 patients were identified. Pathological medical data collected included: histological type, tumor size, modified Scarff Bloom and Richardson (SBR) histopronostic score [25], Ki67 proliferation index, hormone receptor (HR) status including estrogen receptors (ER) and progesterone receptors (PR), epidermal growth factor 2 (HER2) status, and metastatic status. The metastatic status was determined from the initial radiological and histological reports and/or the surgical specimen when available. Tumors were classified according to the latest WHO histological classification [26].

The other medical data collected at the time of histological diagnosis were: age at diagnosis, city of residence, body mass index (BMI), diabetes status, size of the tumor, and presence of metastases. These data were searched in the computerized (CORA software) and paper files of our hospital. When data were missing, the attending physician in charge of the patient was contacted.

### 2.1. Definition of Clinical Groups

Patients were classified into three groups according to their BMI and type 2 diabetes status: control group (non-obese, non-diabetic), diabetes group (type 2 diabetes, obese or non-obese), and obese group (obese non-diabetic).

To determine obese status, BMI was used and classified into four categories according to WHO criteria: thinness: (BMI < 18.5 kg/m^2^), normal weight: (BMI: 18.5–24.9 kg/m^2^), overweight: (BMI 25–29.9 kg/m^2^), and obesity (BMI ≥ 30 kg/m^2^). Obesity include three classes: class 1 (BMI 30–34.9 kg/m^2^), class 2 (BMI 35–39.9 kg/m^2^), and class 3 (BMI ≥ 40 kg/m^2^).

Type 2 diabetes is defined as a blood glucose level at any time of the day ≥2.00 g/L (11.1 mmol/L) or a fasting blood glucose level (i.e., no caloric intake for at least 8 h) ≥1.26 g/L (7.00 mmol/L) tested twice, or blood glucose level ≥2.00 g/L (11.1 mmol/L) measured two hours after 75 g of oral glucose administration [27].

### 2.2. Definition of Molecular Types of Breast Carcinoma

Breast cancers were classified into four molecular types according to their HR expression and HER2 status: two luminal subtypes; luminal A (HR+/HER2) and luminal B (HR+/HER2+); the HER2-enriched type (HR−/HER2+), and the triple-negative (TN) type (HR−/HER2−) [28].

Tumors were considered HR-positive when at least 10% of tumor cells expressed estrogen and/or progesterone receptors by immunohistochemistry (IHC) [29]. Breast cancers were considered HER2 positive according to the criteria defined by ASCO/CAP (American Society of Clinical Oncology/College of American Pathologists) in 2018 [30]: IHC score 3+ (defined as complete and intense membrane staining in >10% of tumor cells), or IHC score 2+ (weak/moderate complete membrane staining in >10% of cells; or intense complete membrane staining in ≤10% of tumor cells) with a dual-probe Fluorescence In Situ Hybridization (FISH) study showing HER2 gene amplification (defined as HER2 gene copy number ≥4 and <6, associated with a ratio of HER2 gene copy number to chromosome17 centromere number ≥2; or HER2 gene copy number ≥6).

### 2.3. GLUT-1 Immunohistochemistry Technique

An automated IHC technique was performed with the Leica Bond Max (Leica Biosystems GmbH, Wetzlar, DE) on 4% formalin-fixed, paraffin-embedded tissue (FFPE). FFPE sections of 3 µm were made with an HM 355S microtome (Microm Microtech France, Brignais, France) and then applied to adhesive slides. A primary monoclonal rabbit anti-GLUT-1 antibody (clone EP141; Epitomics—an Abcam company, Cambridge, UK) diluted at 1/300 was used. The antibody was tested on normal placental and mammary tissue. The rest of the manipulations were performed according to the manufacturer’s recommendations.

### 2.4. Evaluation of GLUT-1 IHC Labeling

**Figure 1:**
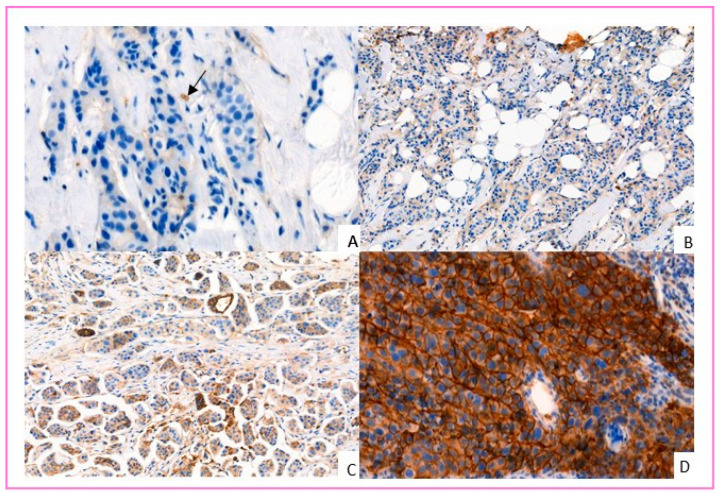
GLUT-1 immunohistochemistry (IHC) score in invasive breast carcinoma. (**A**) Score 0: No membrane staining by tumor cells (positive internal control: red blood cells—black arrow). (**B**) Score 1+: Membrane staining in some tumor cells (<10%). (**C**) Score 2+: membrane staining in ≥10% and <50% of tumor cells. (**D**) Score 3+: membrane staining in ≥50–100% of tumor cells (chicken wire appearance) (IHC GLUT-1, scanned slides, magnification ×330).

GLUT-1 IHC images were obtained by slide scanner (Pannoramic 250, 3D histech, Budapest, Hungary) and interpreted on 3D histech software (caseViewer). Red blood cells were used as an internal positive control. Only membrane staining was considered positive. The evaluation was performed using a semi-quantitative score inspired by Sakashita et al. [31], which grades according to the proportion of GLUT-1 positive cells. We defined a four-grade IHC score: Score 0 negative (no membrane staining by tumor cells); Score 1+, weakly positive (membrane staining in <10% of tumor cells); Score 2+, moderately positive (membrane staining in ≥10 and <50% of tumor cells); Grade 3, strongly positive (membrane staining in ≥50% of tumor cells). Tumors classified as score 2+ or 3+ were considered to overexpress GLUT-1 (Figure 1). All cases were interpreted independently by two pathologists unaware of the patients’ associated medical data. In case of disagreement about GLUT-1 IHC score, cases were discussed until consensus was reached.

### 2.5. Exclusion Criteria

Patients were excluded from this series if they had a diagnosis of recurrent breast cancer, and the primary tumor was not diagnosed in our department. In a second step, patients were excluded for analysis of factors and comparison between the three groups if BMI at diagnosis was not known; and in the third step for GLUT-1 interpretation, if FFPE material was not available. If the patients presented a bifocal or bilateral carcinoma with similar histological and immunohistochemical characteristics, only one location was considered for GLUT-1 expression.

### 2.6. Statistical Analysis

Statistical analyses were performed using Excel software (version 2109, Microsoft Co., Redmond, WA, USA), the biostaTGV website [32] and STATA^®^ software (version 13.1, StataCorp LP., College Station, TX, USA). Categorical, binary, or discrete variables with very few modalities were expressed as headcount and percentage. Quantitative variables that did not reveal a symmetrically shaped distribution were expressed as median, first and third quartile (Q1, Q3), and if relevant, minimum and maximum (min., max.). The χ test^2^ and Fisher’s exact test were performed to test for independence between two categorical variables. The Student’s t-test and the Wilcoxon–Mann–Whitney test were performed to test the independence between a qualitative and a quantitative variable. The statistical tests were two-tailed. The *p* values were considered significant at the 5% level. Missing data were excluded from the statistical analyses.

### 2.7. Ethical Statement

A privacy impact assessment (PIA) and authorization for this study were validated by the Data Protection Officer (DPO) of Cayenne Hospital Center. The non-opposition of the patients was signified by a clinician on the request document sent to the Pathology Department notifying that patient did not object to the use of her sample for scientific research according to the European Data Protection Regulation (GDPR).

## 3. Results

A total of 199 patients were selected; 190 were retained for analysis of general clinical and histological features; 162 for comparative histopronostic analysis of the three groups of patients; and 144 for IHC evaluation of GLUT-1 in these same groups. BMI was missed in 28 patients because 19 patients did not provide either a primary care physician or a telephone number, 8 left a number that was no longer assigned, and one refused to answer questions. Four patients had bifocal carcinoma, three had bilateral carcinoma. Only one specimen from each patient was selected due to similarities in histopronostic and molecular features.

### 3.1. General Clinical and Histological Features of Breast Cancer in French Guiana

**Figure 2:**
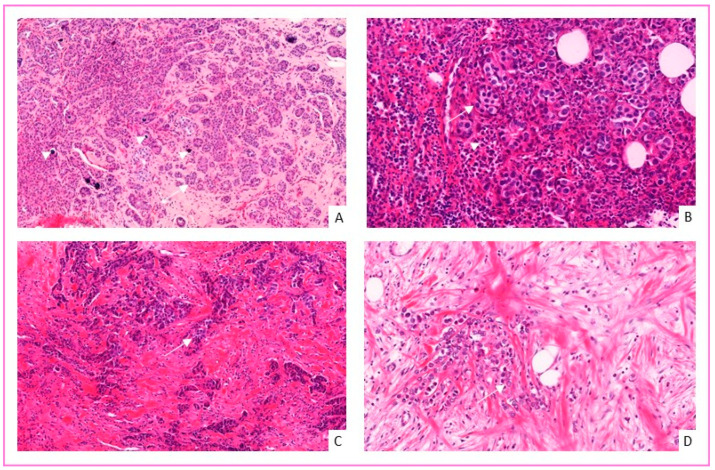
Examples of invasive breast carcinomas in diabetes or obese patients. (**A**) Invasive breast carcinoma NST SBR I shows tubular structures (arrow) consisting of cancer cells with moderate nuclear pleomorphism and low mitotic activity within a sclerotic but low inflammatory stroma (arrowheads: microcalcifications) (magnification ×200). (**B**) Invasive breast carcinoma NST with medullary features shows syncytial cell trabeculae with high nuclear pleomorphism within a TILs-rich stroma (arrowhead) (magnification ×330). (**C**) Invasive breast carcinoma NST SBR II shows no glandular structures (arrow) with moderate nuclear pleomorphism, and moderate mitotic activity, in a sclerotic stroma (magnification ×200). (**D**) Invasive lobular carcinoma shows cells organize in files (arrow) within an edematous stroma (magnification ×400).

Data regarding age at diagnosis, city of residence, histological type, lymph node metastasis, or distant metastasis were available for all patients. BMI, tumor size, modified SBR histological grade, HR status, HER2 status, Ki67 proliferation index, and molecular group were available in 85.3, 69.5, 98.3, 99.5, 97.9, 96.3, and 97.9%, respectively. Clinical and histological data are summarized in Table 1. Figure 2 shows the incidence of breast cancer by the city of residence.

The incidence of breast cancer was highest in the main cities of the territory, where 85% of the population lives; the city of Cayenne and its surroundings (especially Matoury and Rémire-Montjoly), Kourou, and Saint Laurent du Maroni.

The median age at diagnosis was 53.5 years (Q1–Q3: [45.0; 63.0]) (min-max: [25; 92]). The median BMI at diagnosis was 26.6 (Q1–Q3: [23.1; 30.5]). (Additional data regarding age, tumor size and BMI are available in Table 2, Table 3 and Table 4, respectively). The median histological size was 2.8 cm (Q1–Q3: [1.6; 4.1]) (min.max: [0.1; 17]).

There were 57 tumors (30%) with lymph node metastasis at diagnosis and 13 tumors (6.8%) with distant metastasis. Regarding histological types, 172 (90.6%) were invasive carcinomas of no special type (NST), 6 of which were of medullary pattern, 11 (5.8%) invasive lobular carcinomas, 4 (2.1%) invasive papillary carcinomas, one (0.5%) invasive micropapillary carcinoma, one tubular carcinoma, and one mucinous carcinoma (Figure 2). The tumors were of modified SBR histological grade I in 18 (9.6%) cases; grade II in 117 (62.6%) cases; and grade III in 52 (27.8%) cases. HR status was positive in 131 (69.3%) patients, and HER2 was positive in 41 (22.0%) patients. The median Ki67 proliferation index was 20 (Q1–Q3: [10.0;40.0]). The molecular types of the tumors were luminal A in 108 cases (58.1%), luminal B in 21 cases (11.3%), HER2-enriched in 20 cases (10.7%), and TN in 37 cases (19.9%).

### 3.2. Histological and Prognostic Features of Breast Cancer in French Guiana

Of the 162 analyzed cases, 100 were in the control group, 36 in the diabetes group, and 26 in the obese group. Half of the patients in the diabetes group were obese (18/36; 50%). There were no significant differences between clinical groups and the presence of lymph node or distant metastasis at diagnosis, histological grade, HR, and HER2 status, Ki67 proliferation index, and molecular type. We found a trend for the presence of distant metastasis at diagnosis for diabetic patients (*p* = 0.067) and a trend for negative HR status for patients in the obese group (*p* = 0.061) compared to the control group (Table 5 summarizes the results of each analysis).

Data on tumor size, modified SBR histological grade, HR status, HER2 status, Ki67 proliferation index, and molecular type were available in 80, 98.8, 99.4, 99.4, 96.3, and 99.4%, respectively.

### 3.3. GLUT-1 Expression in Breast Cancer Cells

In total, 144 patients with FFPE available material were included for GLUT-1 IHC analysis, 91 were in the control group, 31 in the diabetes group, and 22 in the obese group. Data regarding tumor size, modified SBR histological grade, HR status, HER2 status, Ki67 proliferation index, and the molecular group were available in: 78.5, 99.3, 100, 100, 97.2, and 100%, respectively.

Epithelial and myoepithelial cells of the terminal ductulo-lobular units (TDLUs) never showed overexpression. TDLU cells were either negative (score 0) or slightly positives (1 to 9% positive cells; score 1+).

Our analyses revealed GLUT-1 overexpression (score 2+ or 3+) in 87/144 (60.4%). However, there was no significant difference between GLUT-1 overexpression and clinical groups (Table 6).

**Figure 3:**
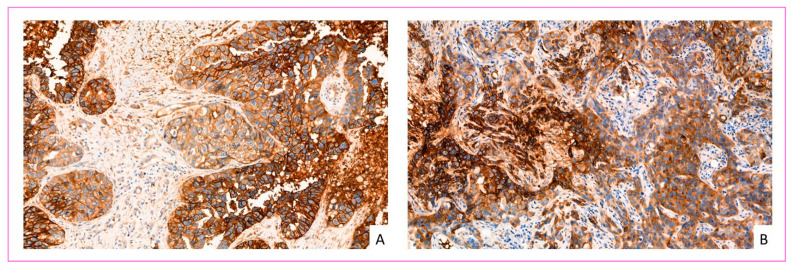
GLUT-1 IHC overexpression in invasive breast cancer cells. (**A**) invasive breast carcinoma NST of triple-negative molecular type, overexpressed GLUT-1 (score 3) (×240 magnification). (**B**) invasive breast carcinomas NST, HER2 enriched molecular type, overexpressed GLUT-1 (score 3) (×300 magnification).

GLUT-1 overexpressed tumors were associated with high SBR histological grade III, higher Ki67 proliferation index, more aggressive molecular types, including HER2 positive and HR negative status (Figure 3). There was no significant correlation between GLUT-1 expression and tumor size or presence of lymph node or distant metastases (Table 7 and Table 8).

Interestingly, all invasive carcinomas NST with medullary pattern presented a strong overexpression of GLUT-1 with IHC score 3+. Some tumors showed heterogeneous expression with areas of high expression and areas of low or no expression.

**Figure 4:**
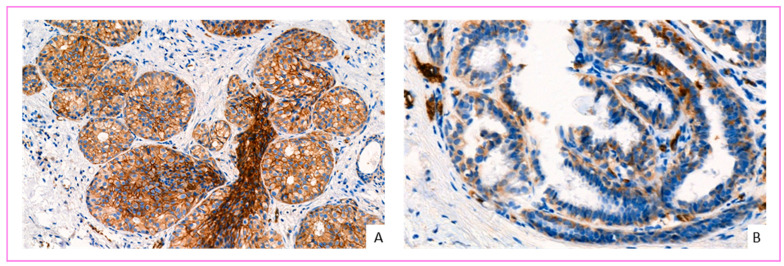
GLUT-1 IHC staining in ductal carcinoma in situ. (**A**) GLUT-1 overexpressed (score 3) in ductal carcinoma in situ of intermediate nuclear grade (magnification ×400). (**B**) Atypical hyperplasia: epithelial cells show weakly membrane staining in <10% of ductal cells (score 1) (magnification ×600).

More interestingly, it was noted that carcinoma in situ always showed GLUT-1 overexpression (*n* = 29) in the three patient groups, with a statistically significant difference compared to associated infiltrative contingent (*p*: 2.60 × 10^−6^, data not shown) (Figure 4A). In contrast, GLUT-1 overexpression was not observed in atypical and non-atypical epithelial hyperplasia (Figure 4B).

We did not find a significant difference for GLUT-1 expression between non-obese diabetic patients and the general population (*p* = 0.718; Table 9), and between obese and non-obese patients regardless of diabetes status (*p* = 0.890; Table 10).

## 4. Discussion

In this study, we described for the first time the clinical, histological, and molecular features of breast cancer in French Guiana. We then compared histological and prognostic factors between patients with and without type 2 diabetes and obesity. Finally, we evaluated the expression of GLUT-1 by IHC technique in these three groups.

French Guiana is inhabited by communities of different ethnic origins, including Creoles and Maroons of African origin, as well as Asian and European populations. Amerindians are the indigenous inhabitants of French Guiana and live mainly in the rainforest along the rivers of the hinterland, maintaining a traditional lifestyle.

The Department of Pathology of Cayenne Hospital Center is the only specialist laboratory in French Guiana, which analyses the majority of samples from the city of Cayenne and adjacent towns, as well as from the isolated communes’ health centers known as CDPS (Centres Délocalisés de Prévention et de Soins). Nevertheless, between 2014 and 2020, we had only 199 patients with invasive breast cancer, which is lower than the reported incidence in French Guiana [33]. In fact, some analyses from hospitals of Kourou and West Guiana in Saint Laurent du Maroni are sent to laboratories in mainland France, resulting in a false lower incidence in our study for these two cities.

In our study, the median age of the patients at the time of diagnosis was 53.5 years, compared to 63 years in mainland France (INCa data), 62 years in the United States of America (USA) [34], and 49 years in Saudi Arabia [35]. Our data corroborate those of a previous study [21]. The young age of the population in French Guiana could at least partly explain these differences. Indeed, the proportion of people in the 60–74 age group is almost three times lower in Guiana than in mainland France (7 and 18%, respectively, INSERM data).

The median tumor size at diagnosis was 2.8 cm. Furthermore, we observed that only 30.3% of the tumors were smaller than 2 cm, which is much lower than what is reported by studies on populations from USA or Poland (58.4 and 51.9%, respectively) [36,37], but closer to the Middle East population of Saudi Arabia (42.6%) [35].

The most frequent molecular group in our population was luminal A (58.1%), followed by TN (19.9%), then luminal B (11.3%), and finally HER2 enriched (10.3%). The frequency of the observed molecular groups was closer to those present in the Saudi population (luminal A: 58.5%, luminal B: 14%, enriched HER2: 11.5%, and TN: 16%) than to those of western populations that report less aggressive TN and enriched HER2 subtypes, in favor of luminal A (such as the USA population: Luminal A: 73.3%, Luminal B: 11.1%, TN: 10.9% and enriched HER2: 4.6%) [35,37,38]. Breast cancer in French Guiana was, therefore, more similar to the Middle Eastern population in terms of molecular type distribution, younger age, and larger size at diagnosis than the Western populations [35].

Our observation reveals an aggressive presentation of breast cancer at diagnosis in French Guiana. This may be due to a delay in diagnosis, suggesting the need to lower the screening age for breast cancer in this territory, as suggested in a previous study [21].

Moreover, we did not observe any significant difference between histopronostic factors and the three patient groups. Nevertheless, there was a trend for negative HR status in the obese group and the presence of distant metastases at diagnosis in the type 2 diabetes group. Our data are different from previous studies that report a lower Ki67 proliferation index and a higher proportion of lymph node metastases in patients with type 2 diabetes [39,40]. This difference may be due to the molecular breast cancer cells specificities of our population which were more aggressive. In agreement with the literature, we did not observe any significant difference between histological SBR grade, histological type, and molecular type in diabetic patients and the control group [40,41].

To our knowledge, this study is the first study that analyzed molecular type in obese patients with breast cancer. In the obese group, we observed a tendency for a negative HR status. The literature on this subject is discordant [42,43].

GLUT-1 is a uniportal transmembrane protein encoded in humans by the *SLC2A1* gene [12,44]. Its main function is to catalyze the entry of glucose into cells [11], and it plays a key role in energy metabolism by providing basal glucose transport in cells. To this end, GLUT-1 is physiologically expressed, at least virtually, in all tissues, and importantly in erythrocytes and endothelial cells of the blood-brain barrier [44]. In this study, we observed that GLUT-1 was overexpressed in 60.4% of the tumors. This overexpression is correlated with high SBR histological grade, high proliferation index, more aggressive molecular types, HER2 positive, and HR negative status. However, this overexpression was not correlated with the obesity or diabetic status of patients.

The overexpression of GLUT-1 in more than 60% of invasive breast cancer in our population is in agreement with the literature, which reports between 35 and 70% of positivity [45,46,47,48,49,50]. The correlation between GLUT-1 overexpression, histological grade, proliferation index, aggressive molecular types, and negative RH status was previously reported [51]. Nevertheless, we observed for the first time an association between the overexpression of GLUT-1 and the positive HER2 status (*p* = 0.020). This result may be related to the molecular specificities of our population.

However, if 60% of breast cancers overexpress GLUT-1, we can hypothesize that cancer cells that do not overexpress GLUT-1 use another metabolic pathway or overexpress other glucose transporters than GLUT-1. Previous studies have shown that GLUT-3, GLUT-4, and GLUT-12 can be upregulated in breast cancer cells [8,9,52].

Surprisingly, all invasive carcinomas NST with medullary pattern showed high GLUT-1 overexpression (score 3+). These carcinomas are characterized by a stroma rich in tumor-infiltrating lymphocytes (TILs) and syncytial carcinomatous cells of high nuclear grade, with high mitotic activity. We hypothesize that increased glycolysis leads to the production of pro-inflammatory metabolites that influence the architecture of the tumor’s inflammatory microenvironment [53]. Alternatively, lactates produced by a shift in metabolism towards glycolysis may be an adaptation of the tumor to escape immune cells [54].

**Figure 5:**
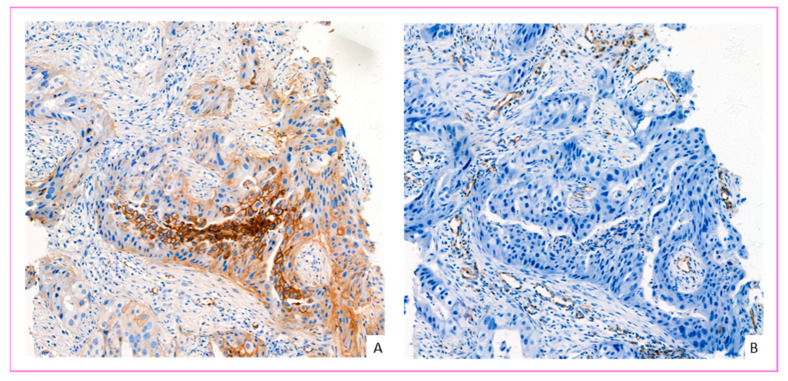
Spatial comparison between GLUT-1 expression and the vascular marker CD31 by immunohistochemistry on the same breast cancer section. (**A**) High-grade ductal carcinoma in situ with invasive area: this figure shows GLUT-1 expression in carcinoma in situ cells (center), which disappears in the invasive lesion (periphery) (**B**). This image shows the neoangiogenesis and vascularization of the tumor stroma by the CD31 endothelial cell marker. The new vessels are located mainly around the invasive carcinoma clusters (magnification ×200).

**Figure 6:**
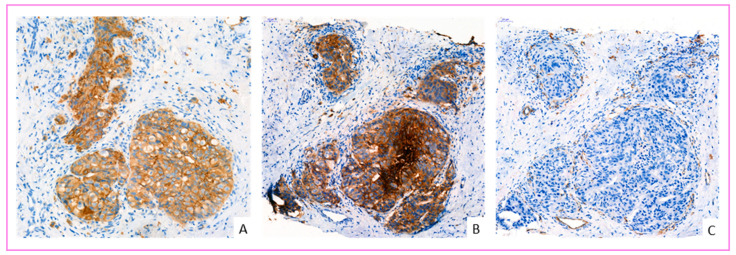
Spatial comparison between GLUT-1 and HER2 overexpressed cancer cells, and the vascular marker CD31 by immunohistochemistry on the same breast cancer section. (**A**) This image shows overexpression of breast cancer cells by HER2 (score 3+). (**B**) The same cancer cells overexpress GLUT-1 (score 3). (**C**) This image shows neoangiogenesis and vascularization of the tumor stroma by CD31. The new vessels are located very close to the invasive carcinoma clusters (magnification ×200).

Interestingly, all carcinoma in situ (CIS) lesions showed GLUT-1 overexpression regardless of their nuclear grade (low grade, intermediate grade, and high grade), but we did not observe overexpression in atypical and non-atypical hyperplasia. This overexpression is independent of the status of adjacent infiltrating carcinoma cells. The absence of expression of GLUT-1 in atypical hyperplasia and the presence of the expression in CIS has been reported previously [49,55]. Furthermore, it has been suggested that this phenotype may be a strong selective advantage for CIS [56]. Our study suggests that a late loss of overexpression of GLUT-1 in breast cancer cells may occur in the invasive stage of tumor progression (Figure 5A), as observed previously with HER2 [57]. However, the spatial comparison using the vascular marker CD31 on the same section revealed the neoangiogenesis of the tumor stroma. The new vessels are located mainly around the invasive carcinoma clusters (Figure 5A). Moreover, a recent study demonstrated that GLUT1 is required for breast cancer formation and robust HER2-induced proliferation even under normoxic conditions [56] (Figure 6A–C). Thus, a further spatial correlation study of GLUT-1 and HER2 can be very informative about the regulation of GLUT1 by HER2.

Furthermore, some tumors showed heterogeneous staining of GLUT-1 with high and low staining areas in the same tumor, suggesting heterogeneity in energy metabolism. This has been highlighted as a property of some tumors [58]. In this study, the expression of GLUT-1 was quantified only at the protein level by immunohistochemistry. However, real-time PCR and digital PCR can give accurate copy numbers and are therefore much more reliable than immunohistochemistry.

We did not find any difference between patient groups and GLUT-1 expression. As many processes are involved in the regulation of GLUT-1, it is possible that other factors such as hypoxia play a prominent role [59]. Hypoxia factor 1a (HIF1a) induces GLUT-1 expression potentially through the PI3K/akt pathway [60]. Indeed, it has been shown that the more distant the cancer cells were from vessels, the more GLUT-1 was expressed [61]. In addition, HIF1a induces intratumoral angiogenesis, reduces mitochondrial metabolism, and promotes glycolysis [60]. Therefore, HIF1a would be involved in the energetic metabolism of cancers [54]. However, the expression of HIF1a in tumors occurs in 50% under conditions of normoxia [62]. This interaction between HIF1a and GLUT-1 could explain some cases of tumor heterogeneity. Thus, further study using hypoxia and vascular markers could be useful to define the oxygenation status of the tumor microenvironment and to investigate the role of hypoxia in GLUT-1 overexpression in breast cancer cells. Moreover, spatial comparison of hypoxia and vascular markers and GLUT-1 can inform if GLUT-1 expression is related to glycolytic phenotype of cancer cells and to obesity/diabetes status of the patients.

Previous studies revealed that in breast tumors, GLUT-1 overexpression on IHC was associated with high-grade histological tumors, with negative HR, TN molecular group, poor total survival, and progression-free survival [45,51,63]. Oh et al. showed that GLUT-1 had a role in TNBC tumorigenesis by inhibiting GLUT-1 and observed a decrease in cell growth, migration, and invasion [64]. GLUT-1 silencing also decreased EGFR activation and the MAPK cascade (c-Raf/MEK/ERK). An involvement in the epithelial–mesenchymal transition due to the observed decrease in CD44—itself correlated with this transition—was also suggested [56]. The deregulation of the energy system is one of the major characteristics of cancers [5]. Several types of regulatory changes have been described [47]. The most common is the Warburg effect [6,7], in which the tumor cell, instead of making energy available through oxidative phosphorylation, will use the aerobic glycolysis pathway, resulting in the formation of lactates and a relatively small amount of available energy (2 moles of ATP/mole of glucose). The tumor cell will therefore have particularly high glucose requirements. This apparent lack of efficiency allows the cell to produce metabolites necessary for its growth and proliferation. The tumor cell captures glucose in large part via GLUT-1 [64]. Thus, GLUT-1 is logically associated with more aggressive tumors [13].

Finally, GLUT-1 overexpression, frequently observed in invasive breast cancer, may present a prognostic factor, and also an interesting therapeutic target, independently of diabetic or obese status. We believe that a further clinical study in a larger cohort with a consensus on the evaluation of GLUT-1 expression in breast cancer cells is needed to determine a score to stratify patients eligible for promising GLUT-1 target therapy.

## 5. Conclusions

We have described for the first time the clinical, histological, and molecular features of breast cancer in French Guiana. The breast cancers in our population are characterized by a young age at diagnosis, aggressive molecular types, and large tumor size. Our results highlight the need to lower the screening age of breast cancer in French Guiana.

We also demonstrated an overexpression of GLUT-1 in 60% of invasive breast carcinomas and in all medullary pattern and carcinoma in situ lesions. Nevertheless, there was no significant difference in GLUT-1 expression between diabetic or obese patients and the control group. The overexpression of GLUT-1 observed in invasive breast cancer and in carcinoma in situ lesions may present a tumor metabolic prognostic marker and also an interesting target therapy, independently of patient metabolic disorder.

## Figures and Tables

**Table 1:** General clinical and histological features at diagnosis (*n* = 190).

Age (year) † (*n* = 190)	53.5 [45.0;63.0]	Modified SBR (*n* = 187)	
BMI (kg/m^2^) † (*n* = 162)	26.6 [23.1;30.5]	I	18 (9.6%)
		II	117 (62.6%)
Tumor size (cm) † (*n* = 190)	2.8 [1.6;4.1]	III	52 (27.8%)
		HR+ * (*n* = 189)	131 (69.3%)
M+ * (*n* = 190)	13 (6.8%)		
N+ * (*n* = 190)	57 (30.0%)	HER2+ * (*n* = 186)	41 (22.0%)
Histological types (*n* = 190)		Ki67 † (*n* = 183)	20 [10;40]
NST	172 (90.6%)	Molecular Groups *	
CLI	11 (5.8%)	(*n* = 186)	
CPI	4 (2.1%)	Luminal A	108 (58.1%)
Tubular	1 (0.5%)	Luminal B	21 (11.3%)
Micro-papillary	1	HER2 enriched	20 (10.7%)
Mucinous	1	TNBC	37 (19.9%)

*: number (%); † median [Q1, Q3]; M+: distant metastasis; N+: regional lymph node metastasis; NST: non-specific invasive breast carcinoma: CLI: invasive lobular carcinoma; IPC: invasive papillary carcinoma; Micro. Pap: invasive micro papillary carcinoma; TNBC: triple-negative breast carcinoma.

**Table 2:** Distribution of patients by age group (*n* = 190).

Age Groups (Years)	Nb. (%)
<35	10 (5.2)
35–49	63 (33.2)
50–64	71 (37.4)
65–74	23 (12.1)
≥75	23 (12.1)

**Table 3:** Tumor size at diagnosis (*n* = 132).

Size (cm)	Nb. (%)
<2 cm	40 (30.3%)
2–5 cm	66 (50%)
>5 cm	26 (19.7%)

**Table 4:** Distribution of BMI in the population (*n* = 162).

Category	BMI (kg/m^2^)	Nb. (%)
Thinness	<18.5	2 (1.2)
Normal weight	[18.5–24.9]	55 (34.0)
Overweight	[25–29.9]	61 (37.7)
Obesity including:	≥30	44 (27.1)
Obesity class 1	[30–34.9]	27 (16.7)
Obesity class 2	[35–39.9]	12 (7.4)
Obesity class 3	≥40	5 (3.0)

**Table 5:** Clinicopathological comparisons of diabetic and obese patients with the control group.

Parameters	Total (*n* = 162)	Control (*n* = 100)	Diabetes (*n* = 36)	*p* Value	Obese (*n* = 26)	*p* Value
Tumor size (cm) †	2.8 [1.8;4.5] (*n* = 129)	2.8 [1.9;4.2] (*n* = 76)	2.5 [1.5;4.0] (*n* = 32)	0.370	3.6 [2.0;7.0] (*n* = 21)	0.378
M+ *				0.067		0.632
Yes	13 (8.0)	5 (5.0)	6 (16.7)		2 (8.3)	
No	149 (92.0)	95 (95.0)	30 (83.3)		24 (91.7)	
N+ *				0.157		0.161
Yes	56 (34.6)	35 (35.0)	8 (22,2)		13 (50.0)	
No	106 (65.4)	65 (65.0)	28 (77.8)		13 (50.0)	
SBR modified *				0.246		0.329
I	11 (6.9)	6 (6.1)	2 (5.6)		3 (12.0)	
II	103 (64.3)	69 (69.7)	20 (55.5)		14 (56.0)	
III	46 (28.8)	24 (24.2)	14 (38.9)		8 (32.0)	
RH *				0.203		0.061
Positive	112 (69.6)	75 (75.0)	23 (63.9)		14 (56.0)	
Negatives	49 (30.4)	25 (25.0)	13 (36.1)		11 (44.0)	
HER2 *				0.904		0.672
Positive	38 (23.6)	24 (24.0)	9 (25.0)		5 (20.0)	
Negative	123 (76.4)	76 (76.0)	27 (75.0)		20 (80.0)	
Ki67 †	20 [20;40] (*n*= 156)	20 [10;35] (*n* = 97)	20 [10;40] (*n* = 35)	0.666	27.5 [15;52] (*n* = 24)	0.082
Molecular Groups * (Molecular Groups)				0.780		0.256
Luminal A	90 (55.9)	59 (59.0)	19 (52.8)		12 (48.0)	
Luminal B	23 (14.3)	16 (16.0)	5 (13.9)		2 (8.0)	
HER2 enriched	16 (9.9)	9 (9.0)	4 (11.1)		3 (12.0)	
TNBC	32 (19.9)	16 (16.0)	8 (22.2)		8 (32.0)	

*: number (%); † median [Q1, Q3]; M+: distant metastasis; N+: regional node metastasis.

**Table 6:** Comparison of GLUT-1 expression between the diabetic and obese patients and the control group.

	Total Nb. (%)	Control Group Nb. (%)	Diabetes Nb. (%)	*p* Value	Obese Nb. (%)	*p* Value
Overexpression of GLUT-1				0.732		0.833
Yes	87 (60.4)	56 (61.5)	18 (58.1)		13 (59.1)	
No	57 (39.6)	35 (38.5)	13 (41.9)		9 (40.9)	
Total	144 (100)	91 (63.2)	31 (21.5)		22 (15.3)	

**Table 7:** Comparison of clinicopathological data with GLUT-1 expression (*n* = 144).

	GLUT-1 Overexpressing	GLUT-1 Not Overexpressed	*p* Value
Size (cm) †	2.5 [2.0;4.6] (*n* = 69)	3 [1.8;5.0] (*n* = 44)	0.594
M+ *			0.344
Yes	5 (5.7)	6 (10.5)	
No	82 (94.3)	51 (89.5)	
N+ *			0.887
Yes	30 (34.5)	19 (51.4)	
No	57 (65.5)	38 (48.6)	
Modified SBR			**0.000420**
I	6 (7.0)	4 (7.0)	
II	49 (57.0)	48 (84.2)	
III	31 (36.0)	5 (8.8)	
RH *			**0.000131**
Positive	50 (57.5)	52 (91.2)	
Negative	37 (42.5)	5 (8.8)	
HER2 *			**0.020**
Positive	27 (31.0)	8 (14.0)	
Negative	60 (69.0)	49 (86)	
Ki67 †	28 [16;60] (*n* = 86)	15 [10;20] (*n* = 54)	**4.51 × 10^−6^**
Molecular Groups *			**2.91 × 10^−6^**
Luminal A	35 (40.2)	47 (82.5)	
Luminal B	16 (18.4)	5 (8.8)	
HER2 enriched	12 (13.8)	3 (5.3)	
Triple negative	24 (27.6)	2 (3.4)	

†: median [Q1, Q3]; *: number (%).

**Table 8:** Comparison of clinicopathological data and GLUT-1 expression between the diabetic and obese patients and the control group.

	GLUT-1 Over.	Total (*n* = 144)	Control (*n* = 91)	Diabetes (*n* = 31)	Value of *p*	Obese (*n* = 22)	Value of *p*
Tumor size (cm) †							
	Yes	2.5 [2.0;4.6] (*n* = 69)	2.8 [1.8;4.1] (*n* = 44)	3.5 [1.7;4.1] (*n* = 15)	0.780	5.2 [3.1;11.5] (*n* = 10)	0.106
	No	3.0 [1.8;5.0] (*n* = 44)	2.5 [1.9;5.0] (*n* = 25)	2.4 [2.0;4.0] (*n* = 12)	0.696	2.6 [2.3;4.9] (*n* = 10)	0.855
M+ *					1		1
	Yes	5 (45.4)	2 (40.0)	2 (50.0)		1 (33.3)	
	No	6 (54.6)	3 (60.0)	2 (50.0)		2 (66.7)	
N+ *					0.677		0.735
	Yes	30	18	3		9	
	No	19	12	3		4	
Modified SBR	Yes				0.270		0.125
I		6	3	2		1	
II		49	36	8		5	
III		31	16	8		7	
	No				0.209		1
I		4	3	0		1	
II		48	30	10		8	
III		5	2	3		0	
HR+ *					0.574		0.254
	Yes	50 (49.0)	37 (52,1)	9 (45.0)		9 (69.2)	
	No	52 (51.0)	34 (47.9)	11 (55.0)		4 (30.8)	
HER2+ *					1		1
	Yes	27 (49.0)	17 (77.3)	6 (75.0)		4 (80.0)	
	No	8 (22.9)	5 (22.7)	2 (25.0)		1 (20.0)	
Ki67 †							
	Yes	28 [16;60] (*n* = 86)	22 [15;54] (*n* = 55)	35 [20;56] (*n* = 18)	0.195	33 [25;65] (*n* = 13)	0.144
	No	15 [10;20] (*n* = 54)	15 [10;20] (*n* = 33)	15 [10;18] (*n* = 13)	0.641	19 [12;38] (*n* = 8)	0.185

GLUT-1 Over. GLUT-1 overexpression; †: median [Q1, Q3]; *: number (%); M+: presence of distant metastasis; N+: presence of regional lymph node metastasis; RH+: RH positive status; HER2+: HER2 positive status.

**Table 9:** Comparison of GLUT-1 overexpression between non-obese diabetic patients and the general population.

	Non-Obese Diabetics No. (%)	Rest of the Population No. (%)	Total No. (%)	*p* Value
				0.718
GLUT-1 overexpressing	9 (56.2)	78 (60.9)	87 (60.4)	
GLUT-1 not overexpressed	7 (43.8)	50 (39.1)	57 (39.6)	
Total No. %	16 (11.1)	128 (88.9)	144 (100)	

**Table 10:** Comparison of GLUT-1 overexpression between obese and non-obese patients regardless of diabetes status.

	Obese No. (%)	Non-Obese No. %	Total No. (%)	*p* Value
				0.890
GLUT-1 overexpressing	22 (59.5)	65 (60.7)	87 (60.4)	
GLUT-1 not overexpressed	15 (40.5)	42 (39.3)	57 (39.6)	
Total No. %	37 (25.7)	107 (74.3)	144 (100)	

## Data Availability

The data used and analyzed during the current study are available in the manuscript text and tables. Further information could be obtained from the corresponding author on reasonable request.
